# Nutrition Modulation of Cardiotoxicity in Breast Cancer: A Scoping Review

**DOI:** 10.3390/nu16213777

**Published:** 2024-11-03

**Authors:** Emma Stephenson, Marie Mclaughlin, James W. Bray, John M. Saxton, Rebecca V. Vince

**Affiliations:** 1 School of Sport, Exercise and Rehabilitation Sciences, University of Hull, Hull HU6 7RX, UKmarie.mclaughlin@ed.ac.uk (M.M.); j.bray@hull.ac.uk (J.W.B.); john.saxton@hull.ac.uk (J.M.S.); 2 Physical Activity for Health Research Centre, Institute for Sport, P.E. and Health Sciences, Moray House School of Education and Sport, University of Edinburgh, Edinburgh EH8 8AQ, UK

**Keywords:** cardiotoxicity, breast cancer, nutrition, antioxidant, LVEF

## Abstract

Background/Objectives: Advancements in breast cancer therapeutics, such as anthracyclines, are improving cancer survival rates but can have side effects that limit their use. Cardiotoxicity, defined as damage to the heart caused by cancer therapeutics, is characterised by a significant reduction in left ventricular ejection fraction (LVEF) and symptoms of cardiac dysfunction. Multiple oral supplements exist with antioxidant and anti-inflammatory properties that have the potential to lower cardiotoxicity risk and ameliorate the complications associated with left ventricular dysfunction. In this review, we evaluate the current status of using nutritional interventions to modulate cardiotoxicity. Methods: We used specific keywords to search for articles that met our predetermined inclusion and exclusion criteria to review the evidence and provide insights for future research. Results: Seven studies were identified as eligible for this review: six focused on oral supplementation strategies in breast cancer patients undergoing chemotherapy, and one focused on nutritional counselling and adherence to the Mediterranean diet in breast cancer survivors’ post-treatment. There was a significantly attenuated reduction in LVEF in five studies that monitored cardiometabolic health, and there were significant improvements in blood serum levels of cardiac biomarkers across all studies. Conclusions: Current evidence suggests that appropriate nutritional interventions, alongside chemotherapy, can modulate the risk of cardiotoxic side effects. This highlights the potential of oral antioxidant supplementation and Mediterranean diet counselling to decrease tertiary cancer therapy costs associated with cardiovascular complications.

## 1. Introduction

Cancer is a group of diseases categorised by an uncontrolled growth of abnormal cells that can start anywhere in the body and is one of the leading causes of death worldwide [1]. Breast cancer remains one of the most prevalent cancers amongst women [2], with 2.3 million women diagnosed across 157 countries annually [3]. The five-year survival rate for breast cancer in the UK is >80%, largely due to improvements in early diagnosis and the recent advancements in breast cancer treatments [4]. Recent developments in cancer therapy and prognosis have significantly improved clinical outcomes, including 5-year survival rates [5]. However, whilst improvements in treatments and prognosis of breast cancer have increased the number of cancer survivors, treatments such as anthracyclines have side effects that limit their use and may subsequently expose patients to increased risk of cardiovascular morbidity and mortality [6]. Cardiotoxicity is defined as toxicity that affects the heart, primarily caused by cancer therapeutics [7]. It is an important adverse reaction to chemotherapy and is associated with increased risk of morbidity, mortality, and early death [8], and it has been identified at follow-up in up to 37.5% of patients undergoing anticancer therapy [9]. Current guidelines suggest that cardiotoxicity in oncology patients may be defined as a post-treatment left ventricular ejection fraction (LVEF) decrease of ≥10% in comparison with LVEF before cancer treatment [10].

Doxorubicin is an anthracycline antibiotic that is commonly administered as a chemotherapeutic agent and is used to treat soft tissue and bone sarcomas and cancers of the breast, ovary, bladder, and thyroid [11]. Despite doxorubicin’s chemotherapeutic efficacy, there are considerable cardiotoxic side effects that undermine the drug’s utilisation as an anti-breast cancer agent [12]. Anthracycline-induced cardiotoxicity is linked to the mitochondrial redox cycling process and primarily arises due to the generation of reactive oxygen species (ROS) via enzyme interactions—doxorubicin generates damage-inducing free radicals in the cardiomyocyte, leading to left ventricular dysfunction [13]. The increased oxidative stress caused by these unstable molecules induces damage to cardiac cells, impairing cardiac function and provoking an inflammatory response, potentially even leading to coronary artery disease and chronic hypertension [14]. As advancements in breast cancer treatment continues to improve, the number of cancer survivors will continue to increase. Because of this, there is growing interest in mitigating a patient’s risk of chemotherapy-related cardiotoxicity without compromising therapy efficacy.

Recent research has highlighted a growing awareness of the potential role of nutrition in diminishing cardiotoxicity [15,16,17]. A variety of nutrients and dietary components have been investigated for their cardioprotective properties, particularly in the context of chemotherapy-induced oxidative stress and inflammation. Antioxidants, such as ginseng, alpha-lipoic acid, vitamin D, and vitamin E, have shown potential in neutralising ROS, which are implicated in anthracycline-induced cardiotoxicity [18,19,20,21]. Polyphenols, found within the Mediterranean diet (fruits, vegetables, whole grains, and healthy fats) and foods like honey, have also been studied for their capacity to reduce oxidative stress and inflammation, thus potentially protecting cardiac tissues from damage [22,23,24]. Furthermore, Coenzyme Q10 (CoQ10), an antioxidant found in foods such as fish, meat, vegetables, fruits, nuts, and oils, has the potential to stabilise mitochondrial function and reduce oxidative damage to cardiac cells during chemotherapy [25]. Emerging research suggests that dietary supplementation, such as vitamin D and ginseng, may modulate inflammatory responses and improve lipid profiles, which are critical in maintaining cardiovascular health in breast cancer patients undergoing chemotherapy [18,19].

Given the growing evidence of the interplay between nutrition and cardiotoxicity, this scoping review assessed the recent literature on the cardioprotective effects of nutrition interventions alongside chemotherapy and trastuzumab regimens in women undergoing or following treatment for breast cancer.

## 2. Materials and Methods

This scoping review was conducted and reported according to the Preferred Reporting Items for Systematic Reviews and Meta-Analyses extension for scoping reviews (PRISMA-ScR) guidelines [26] and the five-stage framework outlined in Arksey and O’Malley [27]. The decision to conduct a scoping review was guided by the objectives of the study and the current state of the literature. Scoping reviews are particularly well-suited to exploring broad topics where the available evidence may be heterogeneous or emerging [28]. Given the relatively nascent state of research on nutritional interventions for chemotherapy-induced cardiotoxicity, this review seeks to map the existing literature, identify key concepts, and uncover gaps in knowledge.

### 2.1. Search Strategy

The databases used for the literature search were the Web of Science (all databases) and PubMed; however, hand-searching and snowballing were also incorporated in the search strategy as other sources for additional records [29]. Specific key terms associated with the scoping review were searched using Boolean operators, including: ‘cardiotoxicity’ OR ‘cardiovascular complications’ AND ‘anthracycline*’ OR ‘cancer treatment’ OR ‘chemotherapy’ OR ‘doxorubicin’ OR ‘radiotherapy’ OR ‘recovery’ AND ‘antioxidant*’ OR ‘diet’ OR ‘nutrition’ OR ‘phytochemical*’ OR ‘supplement*’ OR ‘omega’ or ‘vitamin’ AND ‘breast cancer’. The search was limited to articles published between 2018 and the present day in the English language. The range of years considered in this review was deliberately limited to focus on the most recent advancements in both cancer therapy and nutrition science.

### 2.2. Study Selection

The inclusion criteria were individuals aged 18 years or older with breast cancer, inclusion of a nutritional analysis or an oral nutritional intervention and full text available (with no co-morbidity exclusion). Exclusion criteria included study protocols alone, meta-analysis, systematic reviews, rapid reviews, narrative reviews, in vitro models, and animal models.

All studies were hand-screened according to the search criteria above. During the screening process, duplicates from the two databases were removed, and titles/abstracts were assessed to exclude irrelevant studies. The remaining studies were further assessed, and full-text eligibility was determined according to the inclusion/exclusion criteria. For articles where the full text was not available, the authors were contacted.

**Figure 1:**
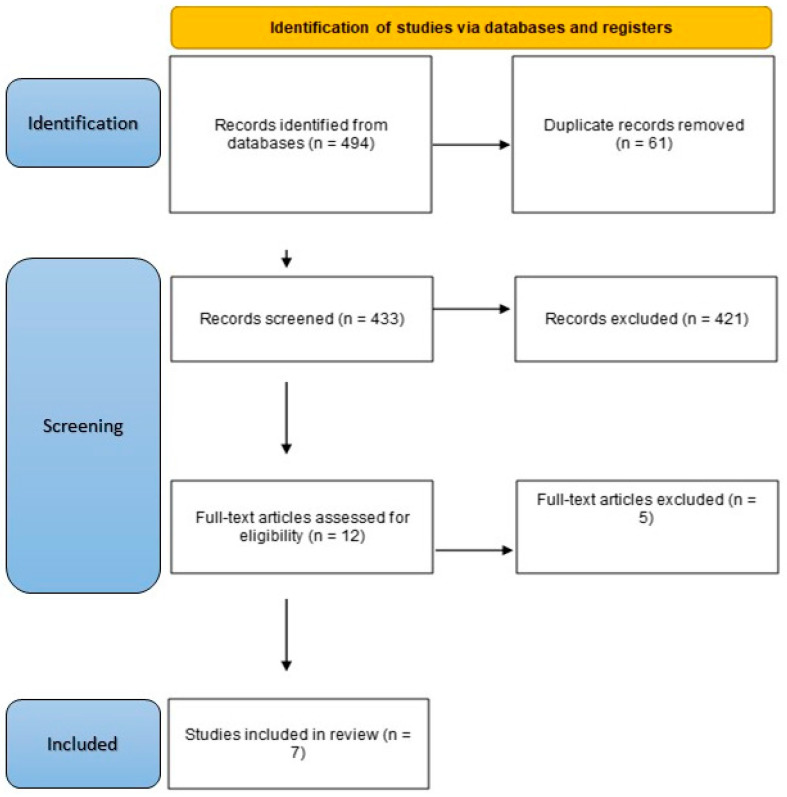
Flow diagram of identification, screening, and inclusion of studies.

The initial search returned 494 papers, of which 61 were duplicates. A total of 433 non-duplicate papers were screened according to the flowchart presented in Figure 1 outlining the step-by-step process of the applied inclusion/exclusion criteria for the literature search strategy. The primary outcome measure was to determine the effects of nutritional interventions on changes in LVEF and cardiac biomarkers, with secondary outcomes including concentrations of blood-borne biomarkers of oxidative stress and inflammation, alongside any effects on body composition, functional mobility, nutritional status, and/or health-related quality of life (HR-QoL). The search strategy was formulated by E.S. and R.V.V. Subsequently, eligibility and conducting the comprehensive analysis for this review were performed by one reviewer (E.S.), who was not blinded to the journal titles or study authors, with any uncertainty resolved through discussion or consultation with R.V.V. and M.M.

### 2.3. Data Analysis

As proposed by [30], articles were inserted into a tabular format to condense and extract the most relevant information for review, whilst allowing for the differentiation of studies and their key themes. The table included study authors, participant characteristics, intervention and control, and key outcomes.

## 3. Results

There were 7 studies included from the 494 reviewed for this scoping review. These studies included a total of *n* = 484 participants, with sample sizes for individual trials ranging from 30 to 150, with a median of 64. All participants were female, aged 18–75 years, with current breast cancer or a previous breast cancer diagnosis. Six of the seven studies were conducted with interventions alongside an adjuvant chemotherapy regimen, while one intervention was conducted in breast cancer survivors that were less than 12 months post-surgery and post-chemo/radiotherapy. The participant characteristics, intervention details, and outcome measures for each study are detailed in Table 1.

### 3.1. Intervention Characteristics

In six of the seven studies, the breast cancer patients received a standard doxorubicin or other undisclosed anthracycline chemotherapy regimen. Nutritional interventions were carried out in these studies as an adjunct to the normal treatment cycles. Intervention duration for these six studies ranged from as short as 14 days (multiflora honey) up to one year (CoQ10), with most study interventions spanning from the start of participants’ chemotherapy treatment until the final cycle of their protocol. A key trend in the nutritional intervention strategies chosen was that all supplementations had some associated antioxidative properties, with a focus on reducing oxidative stress and inflammation.

### 3.2. Primary Outcomes

The primary outcome for the clinical studies was incidence and extent of cardiac dysfunction, as determined by echocardiographic data and cardiac biomarkers. The extent of cardiac dysfunction in these studies was defined by echocardiograph measurements of LVEF, whereby a decline of more than 10% from baseline in a participants’ ejection fraction was considered to indicate cardiotoxicity, with most studies measuring other secondary biomarkers of cardiac/cardiovascular health and one monitoring cardiac events. There was variation in the different outcome measures reported between studies, thus making comparison more challenging, especially given the diversity in terms of the range and duration of nutritional interventions employed, alongside differences in subject characteristics and medical intervention protocols. However, all studies reported at least one improvement, and often more, in terms of changes in ejection fraction and other biomarkers of cardiac dysfunction, including a lower number of cardiac events (Table 1).

Al-Hammadi et al.’s [25] CoQ10 intervention, in conjunction with trastuzumab treatment, mitigated the cardiotoxic effects of chemotherapy. This is evidenced by the maintenance of LVEF in the intervention group compared to the control group, which experienced significant declines in LVEF from baseline to end of the 12-month treatment period. Furthermore, cardiac events decreased significantly in a vitamin E and levocarnitine intervention group compared to standard care controls [31]. In the clinical trial by Hamidian et al. [19], the placebo group had significantly lower LVEF compared to the ginseng group after the eighth chemotherapy cycle (*p* = 0.002). The ginseng group’s mean LVEF values showed no significant changes across three time points (baseline and after the fourth and eighth cycles; *p* = 0.17), while the placebo group exhibited significant declines over the study period (*p* < 0.001). This suggests that ginseng supplementation may help mitigate doxorubicin-induced early declines in LVEF. Additionally, there were no recorded events of cancer therapeutic-related cardiac dysfunction in participants from the ginseng group, while five incidents were recorded in the control group, denoting a difference in the incidence of cardiotoxicity between the two groups (*p* = 0.02). Despite this, no differences in high-sensitivity cardiac troponin I levels were observed. Natalucci et al. [22] also showed no change in high-sensitivity troponin I following a 3-month lifestyle intervention which included Mediterranean diet counselling; however, changes in cardiorespiratory fitness, cardiac function indices, and heart rate variability significantly improved, suggesting cardiometabolic health benefits.

The reviewed studies overall showed significant reductions in serum levels of B-type natriuretic peptide and N-terminal pro b-type natriuretic peptide (NT-proBNP) from baseline in the nutritional intervention groups compared to the control groups. There was a significant pre-test-to-post-test difference in the NT-proBNP delta from baseline (*p* = 0.006) with honey supplementation in the study by Dharma et al. [32], alongside a significant difference between the treatment group and control group troponin I level post-treatment between groups (*p* = 0.031). Vitamin D supplementation resulted in a significant decrease in cardiac troponin T serum levels compared to control group (*p* < 0.001), indicating reduced doxorubicin-induced cardiotoxicity [18]. Al-Hammadi et al. [25] also reported a significant reduction in troponin I levels in the CoQ10 intervention group compared to the control group at all study follow-up time points (*p* < 0.05). Moustafa et al. [31] found that there was no significant increase in serum levels of the cardiac muscle-damage biomarkers troponin I and creatine kinase–myocardial band (CK-MB) from baseline when supplementing vitamin E and levocarnitine as a nutrition intervention, contrasting with a significant increase in CK-MB in the control group. CK-MB levels showed significant elevation after every cycle of chemotherapy in the control group versus the intervention group, indicating a cardioprotective effect. Furthermore, Werida et al. [20] reported that a nutrition intervention with alpha-lipoic acid resulted in a significant decline in serum B-type natriuretic peptide levels compared to the control group (*p* < 0.0001), which may provide a cardioprotective effect on the basis of its antioxidant and anti-inflammatory activities. However, alpha-lipoic acid supplementation had no effect on LVEF compared with the control group. In contrast, in the clinical trial on vitamin E supplementation by Moustafa et al. [31], serum B-type natriuretic peptide levels increased significantly from baseline in both the intervention and control groups (*p* = 0.012 and *p* = 0.006, respectively).

### 3.3. Secondary Outcomes

In general, antioxidant supplementation as part of a nutrition intervention during anthracycline chemotherapy resulted in a significant reduction in oxidative stress and inflammatory biomarkers, which were important secondary (mechanistic) outcomes for many studies. Statistically significant reductions in interleukin-6 (IL-6) were reported when supplementing with both CoQ10 or vitamin D as a nutrition intervention strategy (*p* < 0.05, *p* < 0.001, respectively) [18,25]. Al-Hammadi et al. [25] also reported significant decreases in monocyte chemoattractant protein-1 and soluble toll-like receptor 4 compared with the control group (*p* < 0.05); however, no significant difference in mean F2-isoprostane levels between the groups was observed at any data point. Vitamin D supplementation was also associated with a significant decrease in serum levels of lactate dehydrogenase and IL-6, supporting the cardioprotective effects of vitamin D against doxorubicin-induced cardiotoxicity through attenuation of the pro-inflammatory cytokine induced by doxorubicin and also the potential modulation of oxidative stress [18].

Furthermore, the clinical trial on alpha-lipoic acid supplementation conducted by Werida et al. [20] showed significant reductions in serum levels of an inflammatory marker (TNF-α), an oxidative stress marker (MDA), and neurotensin (involved in modulation of pain signal transmission and perception) in participants in the alpha-lipoic acid group compared with the control group (*p* < 0.0001, *p* = 0.0001, and *p* < 0.0001, respectively). Alpha-lipoic acid also attenuated paclitaxel-associated peripheral neuropathy in women with breast cancer, which could be a consequence of activation of the inflammatory cascade. Of the seven studies included in this systematic review, two studies conducted evaluations on HR-QoL as a secondary outcome measure of intervention efficacy. A 12-item neurotoxicity questionnaire was used to evaluate severity and impact of neuropathy on the patient’s life in the alpha-lipoic acid intervention study conducted by Werida et al. [20], and the intervention group scored significantly higher in comparison with the control group after 9 weeks and 12 weeks of paclitaxel intake (*p* = 0.03 and *p* = 0.004, respectively), with higher scores denoting better HR-QoL. Additionally, after the 3-month intervention conducted by Natalucci et al. [22] in breast cancer survivors, focusing on adopting the Mediterranean diet alongside physical activity, there was a statistically significant reduction in body mass index (BMI), cardiorespiratory fitness, and metabolic and inflammatory parameters. Adherence to a Mediterranean diet was assessed by the MedDiet questionnaire, and adherence to the Mediterranean diet and physical activity levels increased by 28.0% and 61.2%, respectively. The authors also suggested that changes in nutrition and physical activity positively impacted HR-QoL in cancer patients and survivors, although no specific quality-of-life measure was employed.

## 4. Discussion

This scoping review assessed the recent literature on the cardioprotective effects of nutrition interventions alongside anticancer treatments in women undergoing or following treatment for breast cancer. Generally, the findings show promising evidence that antioxidant and anti-inflammatory oral supplementations, alongside adjuvant chemotherapy treatment regimens, may attenuate the risk of cardiotoxicity and its complications.

Nutritional intervention could be a pivotal strategy for managing cardiometabolic health in this context. Nutritional considerations have been reported to reduce inflammation and oxidative stress in clinical studies [33]. There are additional and specific roles of nutritional intervention that accompany curative cancer treatment which have clear potential to improve the response and/or tolerance to cancer therapeutics and reduce the risk of severe side effects and complications [33]. Increasing the consumption of foods with anti-inflammatory properties, like fruit and vegetables, has been quantitatively assessed to be related to a reduced risk of coronary heart disease and is associated with lower mortality from all causes [34]. Additionally, the antioxidant and anti-inflammatory action of carotenoids, and their protective effect against cardiovascular events, are well supported by the literature [35].

Oral supplementation of CoQ10 mitigated cardiomyocyte damage, decreased tissue inflammation, and partially restored normal LVEF compared to the control group [25]. This highlights the possibility of utilising CoQ10 to decrease the cardiotoxic effects of trastuzumab on cardiac muscle, and Al-Hammadi et al. also observed significantly fewer cardiac events in the intervention group. Coenzyme Q10 is integral to energy production within cardiac tissues through its involvement in aerobic respiration and cellular metabolism [36], directly supporting the heart and circulatory system; and CoQ10 deficiency is a commonality in various cases of cardiac dysfunction [37]. Patients with chronic heart failure have notably lower CoQ10 levels, both in blood and myocardial tissue samples [38]. In heart failure, the loss of LVEF is caused by a mitochondrial energy depletion, which is directly associated with low endogenous CoQ10 levels [39].

Reduced CoQ10 levels contribute to cardiomyopathy and myocardial dysfunction [40], and there is significant interest in the therapeutic potential of CoQ10 supplementation, particularly as an adjunct to heart failure treatment [41,42]. Enhancement of ejection fraction is likely due to CoQ10’s capacity to inhibit plasma LDL oxidation and improve endothelial function, coupled with its ability to enhance myocardial bioenergetics [43]. These mechanisms can collectively improve LVEF, which reflects improved cardiac function. In addition, a study on chronic heart failure associated the oral supplementation of CoQ10 with improved functional capacity, endothelial function, and left ventricular contractility [44]. Furthermore, a study on individuals with stable and mild congestive heart failure supported the therapeutic benefits of CoQ10 supplementation previously discussed [45]. Therefore, taken together, these findings establish a rationale that the oral supplementation of CoQ10 could potentially provide cardioprotective support against cardiotoxicity during doxorubicin treatment.

Moustafa et al. [31] found that vitamin E supplementation reduced the number of cardiac events and alleviated the chemotherapy-induced elevations in CK-MB and cTnI levels versus controls. Vitamin E is a potent lipid-soluble antioxidant that plays a critical role in cardiovascular health [46], protecting polyunsaturated membrane lipids from free radical attack and thereby mitigating oxidative damage [47]. Furthermore, vitamin E contributes to the prevention of atherosclerosis by influencing the activity of several enzymes and modulating the expression of involved genes [48]. This decreases the oxidation of LDL cholesterol, which is a key factor in the pathogenesis of cardiovascular disease [49]. In clinical studies, the anti-inflammatory activity of vitamin E has been shown to reduce serum levels of CRP and IL-6, further augmenting its cardioprotective effects [50,51]. Vitamin E’s ability to protect polyunsaturated membrane lipids from free radical attack is likely to be at least partially responsible for attenuation of the usual increase in serum BNP and CK-MB levels following vitamin E and levocarnitine supplementation [31,47]. Furthermore, vitamin E can neutralise ROS during chemotherapy, mitigating oxidative stress and cellular damage in cardiac tissues [21].

In Dharma et al.’s [32] study using multiflora honey as an intervention, the decline in LVEF from baseline was attenuated in the intervention group versus controls, and this was probably attributable to the cardioprotective effects of phenolic compounds. Multiflora honey, derived from the nectar of multiple floral sources, is increasingly recognised for its therapeutic potential in cardiovascular health management [52]. It possesses a strong antioxidant effect and can improve lipid profiles, mitigating risk factors associated with atherosclerosis [53]. Honey is rich in antioxidants, such as phenolic acids and flavonoids, which are acknowledged as having a preventive role against cardiovascular diseases, especially those commonly associated with oxidative stress [23,54]. As such, the antioxidant and cardioprotective derived actions when utilising multiflora honey as part of an intervention alongside chemotherapy are due to the phenolic compounds present, which have been shown to play a preventative role against cardiovascular diseases and heart failure [23,54]. Furthermore, multiflora honey has shown potential in alleviating other side effects of anticancer therapy; it has been reported as effective in the treatment of oral mucositis in head and neck cancer [55,56] and has shown some promise as a therapeutic supplement for the prevention and management of osteoporosis and breast cancer [57].

The use of honey and its active substances can act as anticancer compounds through various mechanisms, with the exact and full mechanisms of these effects still to be fully elucidated. Various studies have shown anti-inflammatory and antioxidant functions can prevent the initiation, promotion, and progression of cancer by affecting on multiple targets, alongside improving the activity of anticancer agents and the QoL in patients undergoing chemotherapy [58]. As such, the integration of multiflora honey into dietary strategies may help support cardiac function and may also play a role in enhancing resilience against the cardiotoxic effects of anticancer therapeutics.

There is promising evidence to support the cardioprotective effects of vitamin D against doxorubicin-induced cardiotoxicity [18]. Vitamin D supplementation significantly decreased cTnT, LDH, and IL-6 serum levels compared with controls, suggestive of cardio-protection against usual chemotherapy damage. Vitamin D plays a crucial role in regulating the cardiovascular system, and a deficiency is associated with increased risk of cardiovascular diseases [59]. Beyond the cardiovascular implications, vitamin D deficiency is linked to increased risks of various malignancies, including breast and bladder cancer, underscoring its role in cellular health and disease prevention [60,61]. Importantly, vitamin D can potentiate the cytotoxic effects of chemotherapeutic agents such as doxorubicin, whereby cancer cells have increased susceptibility to oxidative damage, reducing their resistance and improving tumour suppression [62]. Vitamin D’s active metabolite, 1,25-hydroxy vitamin D, reduces the production of pro-inflammatory cytokine IL-6 via p38 signalling pathways, potentially explaining the significant decrease in the average IL-6 serum concentration found in the intervention group [63,64]. Doxorubicin would otherwise evoke the overexpression of IL-6, as seen in the control group, leading to vascular inflammation and potential cardiovascular complications. Furthermore, in a mouse triple-negative breast cancer model, Lee et al. [65] showed that vitamin D supplementation decreased doxorubicin-induced cardiotoxicity by decreasing ROS and mitochondrial damage. Importantly, this did not decrease the anticancer efficacy of doxorubicin; however, further work is required to ascertain the optimal dosages of vitamin D needed to improve cardiac function without decreasing doxorubicin efficacy.

Therefore, due to the promising role of vitamin D as an anti-inflammatory and for anti-tumour activity, as highlighted previously, there is an established rationale to clinically trial incorporating vitamin D supplementation alongside chemotherapy regimens. Furthermore, vitamin D deficiency and insufficiency are common in patients treated for breast cancer, and deficiency is associated with numerous adverse effects, such as bone loss, arthralgia, and falls [66]. As such, vitamin D status could be considered during treatment to maintain optimal levels to minimise the risk of treatment-related problems, alongside vitamin D’s ability to potentiate the cytotoxicity of chemotherapeutic drugs, thus potentially improving overall treatment efficacy, demonstrating a potential benefit for this nutritional strategy [62].

Ginseng supplementation also presented promising results regarding protection against doxorubicin-induced early cancer therapeutics-related cardiac dysfunction and early decline in LVEF in breast cancer patients [19]. Ginseng, the root of a Panax ginseng C. A. Mayer plant, is one of the most popular traditional herbal remedies worldwide [67], recognised for its potential to enhance cardiovascular health [68]. Ginseng is rich in several bioactive compounds, with ginsenosides being the primary active components responsible for pharmacological benefits in inflammatory conditions [69]. Ginsenosides have been shown to improve vascular endothelial function through the modulation of myocardial oxidative stress and could potentially help ameliorate the decline in LVEF in cardiovascular diseases [70]. Whilst Hamidian et al.’s study [19] demonstrated high intervention adherence and that ginseng was well tolerated, further work is clearly warranted, including intervention studies in breast cancer patients with a history of cardiovascular disease.

In Werida et al.’s study [20] utilising oral alpha-lipoic acid supplementation, the results showed a significant improvement in serum biomarkers of oxidative stress and inflammation. Alpha-lipoic acid is a powerful antioxidant supplement that functions to mitigate oxidative stress, which is otherwise a key contributor to cardiovascular disease and other chronic diseases [71]. The main mechanisms of antioxidant action associated with alpha-lipoic acid are the scavenging and neutralisation of a variety of ROS [72]. The potential cardioprotective effects of alpha-lipoic acid are particularly relevant in oncology, where the protection of cardiac mitochondrial cells can help to prevent myocardial dysfunction, which is a complication often related to chemotherapy. However, this intervention did not produce a significant effect on LVEF versus controls [20]. Therefore, further investigation is required to determine the cardiac implications of alpha-lipoic acid-induced alleviation of oxidative stress and inflammation.

The 3-month home-based lifestyle intervention on breast cancer survivors improved cardiometabolic health and autonomic function and reduced echocardiographic signs of diastolic dysfunction, alongside improvement in BMI, cardiorespiratory fitness, metabolic, and inflammatory parameters, even in a home-based setting [22]. The intervention strategy included remote delivery of motivational interviews, Mediterranean diet counselling, and supervised aerobic exercise. The Mediterranean diet is characterised by high consumption of fruits, vegetables, whole grains, legumes, nuts, and olive oil, with moderate intake of fish, poultry, and moderate alcohol [73]. Adherence to the Mediterranean diet has been widely acclaimed for its health benefits; reports have associated a higher Mediterranean diet score with lower incidence of cardiovascular disease and cancer [74]. However, in this setting, strategies to improve adherence further may prove advantageous. Comprehensive and repeated research has been conducted to assess the Mediterranean dietary pattern for its cardiovascular effects; the polyphenol-rich foods in this diet have been shown to significantly lower inflammatory biomarkers IL-6 and TNF-α in clinical trials [24], and the study by [22] highlights the potential for implementing this form of intervention alongside breast cancer treatment and for cancer tertiary care. However, similar to all the interventions included in the review, larger trials with longer-term follow-up is required.

There are several risk factors associated with increased risk of anthracycline-induced cardiotoxicity which should be considered, including hypertension, diabetes mellitus, pre-existing heart conditions, and obesity, potentially causing nearly double morbidity of cardiotoxicity [75]. These risk factors should also be considered, and additional nutritional strategies may also have a role in managing this risk during and after anthracycline treatment. Previous research has also developed a cardiovascular disease and cardiotoxicity risk assessment questionnaire to quantify the potential extent of risk factors in breast cancer patients prior to treatment [76], and combined strategies to identify and modulate risk have important future potential. That being said, whilst nutritional assessment and intervention have a place on the cancer continuum of prevention, during treatment, management of side effects, and in preventing a recurrence, we need to be mindful that high doses of certain antioxidants could interfere with the cytotoxic effects of chemotherapy or cause adverse interactions, which must be considered [77,78,79]. As such, there are still inconsistencies within the literature, and clinical teams and patients need to balance lessening side effects and improving quality of life whilst maintaining effective treatment [79]. Ambrosone et al. [80] analysed secondary data comparing chemotherapy schedules in breast cancer and identified an increased hazard of recurrence in women using antioxidant supplements both before and during chemotherapy. Therefore, ensuring patient safety and treatment efficacy is paramount and highlights the importance of further work in the field.

Key limitations of the included studies are the low sample sizes utilised in the interventions and controls groups. There are numerous differences in study protocols in terms of the diversity of nutritional interventions, duration of intervention, and dosing strategy employed, alongside differences in treatment regimen and participant characteristics. The heterogeneity of the studies included within the review is an important limitation that makes generalised conclusions challenging. Because of the varied nature of the different nutritional interventions, it is difficult to draw definitive conclusions about which interventions are most effective, alongside ascertaining what the optimal dosage would be, the ideal length of intervention, and also the timing for implementing these strategies. A further limitation was the exclusive use of studies presented in the English language, and there may be relevant studies published in other languages. It is worth noting that this was a scoping review aimed at addressing a broader topic with different study designs to explore the extent, range, and nature of recent research activity and identify gaps in the existing literature [27]. Previous systematic reviews on this topic do exist [81], although our review builds further on this work, with the inclusion of novel supplements which were not reviewed previously by Zhang et al. (namely vitamin D, ginseng, alpha-lipoic acid, and the Mediterranean diet). Additionally, we include vitamin E and honey supplementation interventions in human rather than in animal models. Therefore, the current review provides a sharper focus on in-human research and offers an up-to-date review building on the initial evidence from previous work in the field.

Furthermore, the interventions were of short duration for the most part, and therefore larger-scale trials are required with a longer-term follow-up, alongside diversification of the subject groups to expand the heterogeneity of participants in terms of stage/grade, treatment, co-morbidities, and age. In an emerging field, there are numerous avenues for further research that is required to standardise future studies to enhance knowledge within the field, alongside a need to more fully elucidate the more mechanistic aspects. There is a need for larger, long-term studies to evaluate the effectiveness of the interventions and also make a comparison between supplements. Qualitative research should also be undertaken in longer-term studies to determine how easy or difficult participants find it in terms of making dietary changes to identify any particular barriers in this population. Longer-term studies are particularly important, as cardiomyopathy can develop in some patients months to years after termination of treatment. Further research is required to better understand the efficacy of these interventions on patient cardiometabolic health, incidence of cardiac dysfunction, the sustainability of cardioprotective effects, five-year survival rates, and the impact on overall cardiovascular health in cancer survivors. Furthermore, larger multicentre randomised controlled trials are required to establish the efficacy of nutritional interventions as a standard adjunctive therapy. Despite these limitations, it is promising to see that there are potential nutritional interventions that are both easily accessible and relatively easy to implement (which, in turn, may facilitate higher compliance), and that are also potentially cost-effective on a wider scale, that could be used as part of treatment pathways to modulate cardiotoxicity and improve cardiovascular risk in breast cancer patients.

## 5. Conclusions

This scoping review showed that there is some potential for suitable nutritional intervention alongside chemotherapy that may be capable of modulating the risk of cardiotoxicity through cardioprotective mechanisms of action. Cardiovascular health is important for breast cancer patients undergoing anthracycline chemotherapy regimens, which are associated with cardiotoxic side effects. The current evidence shows that nutritional supplements with antioxidant and anti-inflammatory properties can significantly improve markers of inflammation and oxidative stress. These improvements, in some cases, were significantly associated with a smaller decline in LVEF and a mitigated risk of cardiovascular dysfunction. As such, nutritional supplementation, which includes antioxidants alongside cancer therapy, does show some promise in terms of benefits beyond cardioprotective effects, such as improved targeted cytotoxicity of cancer treatments. However, further research is clearly warranted to glean further insights into the efficacy of nutritional interventions for improving cardiometabolic health in women undergoing chemotherapy treatments for breast cancer and also survivorship. This future work is imperative to be able to inform standardised guidelines on the type, dosage, and duration of nutritional interventions in a diverse range of patient populations and to be able to translate research findings into practical clinical recommendations. In conclusion, this review has shown the emerging potential of utilising suitable nutritional interventions alongside chemotherapy to modulate the risk of cardiovascular complications. Incorporating nutritional counselling into oncology practice may provide more holistic patient care, with a potential to reduce healthcare costs by preventing or managing cardiotoxicity more effectively.

## Figures and Tables

**Table 1:** Characteristics of eligible studies included in the review.

Year/ Study	Participant Characteristics	Cancer Diagnosis and Therapy	Supplementation	Method and Intervention	Outcomes
[25] Al-Hammadi 2023	Females (*n* = 100). Age not specified. Excluded: under 18 years, pregnant women, and those with congestive heart failure, diabetes, statin treatment, stimulants, thyroid dysfunction, or current anticoagulation.	Breast cancer HER2 + ve (HER2 + 3 or amplified gene) Adjuvant chemotherapy: 4 cycles of Adriamycin (60 mg/m^2^), cyclophosphamide (600 mg/m^2^), followed by 4 cycles of Taxotere (docetaxel 75 mg/m^2^) and Herceptin (trastuzumab 8 mg/kg) loading dose after 3 weeks of rest, and then 6 mg/kg maintenance every 3 weeks, for 17 cycles.	200 mg Coenzyme Q10 (CoQ10) oral capsules twice daily for a year.	Patients allocated to control group (*n* = 50) who received trastuzumab and placebo for a year vs. treatment group (*n* = 50) received Herceptin and 200 mg CoQ10 oral capsules twice daily for a year. Serum samples were collected immediately post treatment at baseline, 3, 6, 9 and 12 months. Cardiac troponin I (cTnI), interleukin-6 (IL-6), monocyte chemoattractant protein-1 (MCP-1), soluble toll-like receptor 4 (sTLR4), and F2-isoprostane were measured by enzyme-linked immunosorbent assay (ELISA). Multigated acquisition (MUGA) scans were used to take images of the heart at various points in time to record and view multiple cardiac cycles.	Highly significant decrease in mean MCP-1 level for CoQ10 group at 3 and 12 months (*p* = 0.001). Significant reductions in MCP-1 at baseline, 6 months, and 9 months (*p* = 0.05, 0.042, and 0.023, respectively). Significant reduction in IL-6 levels at all stages for CoQ10 group vs. control (*p* < 0.05). Highly significant decrease in sTLR4 levels at all stages for CoQ10 group vs. control (*p* = 0.001). No significant difference in F2-Isoprostane (ISO_PGF2α) levels. Significant reduction in cTnI levels for CoQ10 group vs. control at 3 months (*p* = 0.05), and highly significant decrease at baseline, 6, 9, and 12 months (*p* = 0.018, *p* = 0.008, *p* = 0.010, and *p* = 0.008, respectively). Highly significant decrease in the reduction in MUGA scan ejection fraction levels after 3, 9, and 12 months (*p* = 0.001). Significant reduction observed at 6 months (*p* = 0.043), but no significant effect on ejection fraction levels at baseline.
[31] Moustafa 2024	Females (*n* = 74). Aged 52 ± 11 years Performance status of 0–2 in the Eastern Cooperative Oncology Group score, and a LVEF of >50%. Excluded: pregnant women, pre-existing cardiac disease or risk-factors, have previously undergone anthracycline treatment, or had any abnormal baseline bloodwork.	Breast cancer patients: Stage I (*n* = 4) Stage II (*n* = 34) Stage III (*n* = 21) Stage IV (*n* = 15) ER+ (*n* = 53) PR+ (*n* = 49) HER2+ (*n* = 23) Doxorubicin (60 mg/m^2^) and cyclophosphamide (600 mg/m^2^) regimen once every 21 days for 4 cycles.	Vitamin E (600 mg three times daily) and oral levocarnitine (300 mg four times daily) for duration of treatment.	Participants were randomised to a control group (*n* = 39) who received the chemotherapy regimen only vs. an intervention group (*n* = 35) who received a combination of vitamin E and oral levocarnitine alongside chemotherapy. Blood serum cardiac biomarker levels, including B-type natriuretic peptides (BNPs), creatine kinase–myocardial band (CK-MB), and cTnI, were tested. Patient cardiac function was assessed by measurement of LVEF.	The intervention group reported 5 cardiac events during the study, while 15 were reported from the control group. There was a statistically significant difference in the number of cardiac events between groups (*p* = 0.02). In both groups, the median serum BNP levels increased significantly between the baseline and the mean of four cycles (intervention, *p* = 0.012; control, *p* = 0.006). In the intervention group, there was no statistically significant difference between the baseline and the mean of four cycles for both the CK-MB and cTnI level. In the control group, there was a statistically significant difference in CK-MB and cTnI level between the baseline and mean of four cycles (*p* = 0.0001 and *p* = 0.007, respectively).
[32] Dharma 2022	Females (*n* = 36). Aged 53 ± 8 years. Karnofsky index > 70. Excluded: patients who underwent radiotherapy, have several systemic diseases before chemotherapy, are malnourished, are diabetic, used another derivative during chemotherapy, or are allergic to honey.	Invasive ductal breast cancer Grade II (*n* = 28) Grade III (*n* = 8) Fluorouracil–Adriamycin–cyclophosphamide (FAC) chemotherapy (details of dosage omitted)	90 mL/day honey for 14 days.	The experimental study had a double-blind, randomised pre- and post-study design with control group. Participants were randomly allocated into a control group (*n* = 18) who received only FAC chemotherapy vs. treatment group (*n* = 18) who were given FAC chemotherapy and 90 mL/day honey for 14 days. Independent variables measured in this study were troponin I and N-terminal pro b-type natriuretic peptide (NT-proBNP) levels. Data were also collected on patient age, body mass index (BMI), body surface area (BSA), nutrition status, chronic heart function disorders, kidney function disorders, radiation, and breast cancer stage.	There was a significant difference in cTnI levels at the post-test stage between the group treated with honey vs. control group (*p* = 0.031). A significant decrease in the mean post-test NT-proBNP levels in the treatment group (from 461.0 to 215.6). Insignificant increase in the mean post-test NT-proBNP levels in control group (from 275.9 to 315.4). A significant difference in the NT-proBNP delta between the treatment and control group (*p* = 0.006).
[18] El-Bassiouny 2022	Females (*n* = 150). Aged 49 ± 6 years. Good performance status according to the Eastern Cooperative Oncology Group with a score of 0–2, had normal renal and liver function, and a normal baseline Echocardiography. Excluded: pregnant or breastfeeding, had a history of breast cancer, previously underwent doxorubicin treatment, or had an allergy to doxorubicin or vitamin D.	Breast cancer Stage IIa (*n* = 61) Stage IIb (*n* = 39) ER+ (*n* = 90) PR+ (*n* = 90) HER2+ (*n* = 4) Four cycles of adjuvant chemotherapy: doxorubicin (60 mg/m^2^) and cyclophosphamide (600 mg/m^2^). Cycles were repeated every 21 days.	0.5 µg of vitamin D administered orally once daily for duration of treatment.	Participants were randomised into a control group (*n* = 50) who received four cycles of adjuvant chemotherapy vs. a vitamin D group (*n* = 50) who received the same treatment plus 0.5 µg of vitamin D administered orally once daily for the whole treatment course. Blood samples were taken, and kidney and liver function tests were conducted at routine follow-ups prior to each cycle. Serum levels of vitamin D, lactate dehydrogenase (LDH), cardiac troponin T (cTnT), and IL-6 were assessed at baseline and at the end of the study after four treatment cycles.	There was a significant increase in the serum levels of vitamin D in the vitamin D group vs. control group (*p* < 0.001). The vitamin D group showed a statistically significant decrease in cTnT serum levels compared to control group (*p* < 0.001). The percentage change in serum LDH levels was significantly decreased in the vitamin D group compared to the control group (*p* < 0.001). The serum IL-6 levels were significantly decreased in the vitamin D group vs. control (*p* < 0.001). The percent change in vitamin D showed a negative significant correlation with serum levels of LDH, IL-6, and cTnT (*p* < 0.001; r = −0.782, −0.741, and −0.718, respectively).
[19] Hamidian 2023	Females (*n* = 30). Aged 44 ± 1 years. LVEF ≥ 50%. Excluded: patients previously treated with anthracycline-based chemotherapy or radiotherapy, metastatic disease, HER-2+ breast cancer, history of cardiovascular diseases, pregnancy, or breastfeeding.	Non-metastatic breast cancer with HER-2 negative tumour status Anthracycline based chemotherapy regimen (four cycles of doxorubicin (60 mg/m^2^) plus cyclophosphamide (600 mg/m^2^) every 21 days in patients receiving adjuvant chemotherapy (*n* = 17) and every 14 days in patients receiving neoadjuvant chemotherapy (*n* = 13), followed by four cycles of docetaxel (100 mg/m^2^) every 21 days in both groups.	1 g/day ginseng capsules for duration of treatment.	Patients were randomly allocated into a control group (*n* = 15) and a ginseng treatment group (*n* = 15). All participants received a standard anthracycline-based chemotherapy regimen. Patients in the ginseng group received 1 g/day ginseng capsules. The control group received placebo capsules. The intervention spanned from the same day as the first doxorubicin treatment until a week after completion of treatment. Assessment of transthoracic echocardiogram and high-sensitivity cTnI levels was performed at baseline. Participant cTnI levels were measured again after the completion of the final treatment cycle. To measure transthoracic echocardiogram, participants underwent echocardiography at baseline, after the final cycle, and subsequently six months after chemotherapy initiation.	At baseline, there was no significant difference in mean LVEF values between the two groups. In the intervention and control groups, LVEF decreased from 62.0 ± 0.9% to 60.7 ± 1.0% and from 63.27 ± 1.1% to 58.0 ± 1.3%, respectively (difference = 3.97%; *p* = 0.06), between baseline and the end of the fourth cycle of chemotherapy. However, the overall decline in LVEF from baseline to the fourth cycle of chemotherapy was 1.3 ± 1.1% in the ginseng group and 5.27 ± 0.8% in the placebo group (*p* = 0.006). After the eighth cycle of chemotherapy, the control group had significantly lower LVEF than the ginseng group (55.9 ± 1.5% vs. 62.8 ± 1.0%, respectively, *p* = 0.002). Additionally, from baseline to after the eighth cycle, the control group had a 7.3 ± 1.4% reduction in mean LVEF value, while the mean LVEF value increased by 0.8 ± 1.3% for the ginseng treatment group (*p* < 0.001). After the eighth cycle of chemotherapy, five patients from the placebo group (33%) developed cancer therapeutics-related cardiac dysfunction, while no patients from the ginseng group fulfilled the criteria, with a statistically significant difference in the incidence of cardiotoxicity between the two groups after the eighth cycle of chemotherapy (*p* = 0.02). High-sensitivity cTnI levels were not significantly different between groups.
[20] Werida 2022	Females (*n* = 64) Aged 49 ± 8 years. Included patients with Eastern Cooperative Oncology Group performance status <2 with adequate hematologic parameters, liver function, and renal function. Excluded patients with prior exposure to anthracyclines and neurotoxic agents in the last 6 months, evidence of metastasis at initial assessment, concomitant use of antioxidant vitamins, presence of clinical evidence for severe cardiac illness, pre-existing neuropathy, pregnant, or breastfeeding. Also excluded HER2+ candidates.	Breast cancer Stage II (*n* = 33) Stage III (*n* = 31) Four cycles of doxorubicin (60 mg/m^2^) and cyclophosphamide (600 mg/m^2^) every 21 days, followed by weekly doses of paclitaxel (80 mg/m^2^) for 12 weeks.	600 mg alpha-lipoic acid once daily for 6 months.	Participants were randomised into a control group (*n* = 32) and an alpha-lipoic acid group *(n* = 32). All candidates received the same anticancer treatment. The control group received placebo tablets, while the intervention group received alpha-lipoic acid once daily for 6 months. Demographic data were collected from all participants, including age, medication history, physical examination, and BMI. A 12-item neurotoxicity questionnaire from the validated FACT/GOG-Ntx-12 was used to evaluate the severity and impact of neuropathy on patients’ life, where a higher score represents better quality of life. Blood samples were collected 1 h before the first chemotherapy cycle, 1 h after the last anthracycline cycle, and 1 h after the last paclitaxel cycle. Blood serum levels of BNP, tumour necrosis factor-alpha (TNF-α), and neurotensin (NT) were determined by sandwich ELISA. Malondialdehyde (MDA) was determined and used as a marker of oxidative stress. Echocardiographic assessment of chemotherapy-related cardiotoxicity was performed at baseline and after the last doxorubicin/cyclophosphamide cycle.	Both supplementation and control groups showed significant declines in ejection fraction after intervention (*p* < 0.0001 and *p* < 0.0001, respectively), with no significant variation between the two study groups after treatment (*p* = 0.113). The alpha-lipoic acid group showed a significant decline in BNP, TNF-α, MDA, and NT serum levels as compared to the control group (*p* < 0.0001, *p* < 0.0001, *p* = 0.0001, and *p* < 0.0001, respectively). The percentage of patients with grade 3 peripheral sensory neuropathy was significantly lower in the intervention group as compared to the control group (6.3% vs. 25%, respectively, *p* = 0.039). After the 9th and 12th week of paclitaxel cycles, the FACT/GOG-Ntx-12’s total score was significantly higher in the intervention group vs. the control group (*p* = 0.03 and *p* = 0.004, respectively).
[22] Natalucci 2021	Non-physically active females (*n* = 30) Aged 54 ± 8 years. <12-month post-surgery and post-adjuvant chemo/radiotherapy. Included patients’ stage from 0 to III breast cancer without metastases or recurrences diagnosis at recruitment in follow-up. Excluded candidates that had any illnesses that prevented exercise performance, treatment with drugs that have potential effect on heart rate response to exercise (e.g., beta blockers and amiodarone), or treatment with antidepressant drugs.	Breast cancer survivors with high risk of reoccurrence. Stage 0 (*n* = 6) Stage I (*n* = 15) Stage II (*n* = 9) Mastectomy (*n* = 3) Quadrantectomy (*n* = 26) Lumpectomy (*n* = 1) Only radiation (*n* = 2) Only chemotherapy (*n* = 13) Radiation and chemotherapy (*n* = 4) None (*n* = 11) Current endocrine therapy: Tamoxifen (*n* = 8) Aromatase inhibitor (*n* = 16)	Mediterranean diet, remote delivery of motivational interviews, and supervised aerobic exercise.	Participants were randomly allocated to a 3-month intervention arm and a control arm. Forced changes in study protocol due to COVID-19 restrictions made the difference between interventions negligible, providing similar adaptations between groups. All participants received a 3-month lifestyle intervention, including remote delivery of motivational interviews, Mediterranean diet counselling, and supervised aerobic exercise. Participants from both arms were combined into a unique group because the intervention and control trend over time showed no significant differences and changes in all samples were assessed using a general linear model. Participants were assessed before and after the intervention: anthropometrics and body composition; dietary habits and physical activity; cardiorespiratory fitness; and cardiovascular, metabolic, hormonal, and inflammatory parameters.	Post-intervention, there were significant improvement in BMI (pre 26.0 ± 5.0 vs. post 25.5 ± 4.7; −1.7%; *p* = 0.035), reduction in body weight (pre 67.1 ± 11.6 kg vs. post 66.3 ± 10.9 kg; −1.2%). Waist circumference and fat mass did not change. Post intervention, participants had a significantly higher *V*O_2max_, physical activity levels, and adherence to Mediterranean diet (*p* < 0.001, *p* < 0.001, *p* < 0.001, respectively). After the intervention there were improvements in signs of diastolic dysfunction (pre-intervention present in 15 patients vs. 10 patients post with at least one sign; *p* = 0.007) and no subject in the normal range at pre intervention showed new signs of diastolic dysfunction after 3 months. A significant reduction in mean heart rate was found after 3 months (*p* = 0.003). There were significant reductions in glycaemia, insulin, homeostasis model assessment (HOMA), low-density lipoprotein (LDL), cholesterol, testosterone, and high-sensitivity C-reactive protein (CRP) (*p* < 0.001, *p* = 0.018, *p* = 0.005, *p* < 0.001, *p* = 0.029, *p* = 0.003, and *p* = 0.027, respectively). Changes in triglycerides, high-density lipoprotein, progesterone, and high-sensitivity troponin were not significant.

HER2 (human epidermal growth factor receptor 2), Adriamycin (doxorubicin), cyclophosphamide (cyclophosphamide), Taxotere (docetaxel), Herceptin (trastuzumab), CoQ10 (coenzyme Q10), MCP-1 (monocyte chemoattractant protein-1), IL-6 (interleukin-6), sTLR4 (soluble toll-like receptor 4), cTnI (cardiac troponin I), ISO_PGF2α (F2-isoprostane), MUGA (multigated acquisition), LVEF (left ventricular ejection fraction), BNP (B-type natriuretic peptide), CK-MB (creatine kinase–myocardial band), NT-proBNP (N-terminal pro B-type natriuretic peptide), LDH (lactate dehydrogenase), cTnT (cardiac troponin T), GOG-Ntx-12 (Gynecologic Oncology Group Neurotoxicity Questionnaire), MDA (malondialdehyde), TNF-α (tumour necrosis factor alpha), CRP (C-reactive protein), HOMA (homeostasis model assessment), LDL (low-density lipoprotein), HDL (high-density lipoprotein), *V*O_2max_ (maximal oxygen consumption).

## Data Availability

No new data were created or analysed in this study. Data sharing is not applicable to this article.
